# Comprehensive Genomic Profiling of Androgen-Receptor-Negative Canine Prostate Cancer

**DOI:** 10.3390/ijms20071555

**Published:** 2019-03-28

**Authors:** Renée Laufer-Amorim, Carlos Eduardo Fonseca-Alves, Rolando Andre Rios Villacis, Sandra Aparecida Drigo Linde, Marcio Carvalho, Simon Jonas Larsen, Fabio Albuquerque Marchi, Silvia Regina Rogatto

**Affiliations:** 1 Department of Veterinary Clinic, School of Veterinary Medicine and Animal Science, São Paulo State University-UNESP, Botucatu 18680-970, Brazil; sandradrigo@gmail.com (S.A.D.L.); marcio.carvalho@unesp.br (M.C.); 2 Department of Veterinary Surgery and Anesthesiology, School of Veterinary Medicine and Animal Science, São Paulo State University-UNESP, Botucatu 18680-970, Brazil; carlos.e.alves@unesp.br; 3 Department of Genetics and Morphology, Institute of Biological Sciences, University of Brasília-UnB, Brasília 70910-900, Brazil; andrevillacis@yahoo.com.br; 4 Department of Mathematics and Computer Science, University of Southern Denmark, 5230 Odense, Denmark; sjlarsen@imada.sdu.dk; 5 International Research Center (CIPE), A.C. Camargo Cancer Center, São Paulo 01508-010, Brazil; fabio.marchi@accamargo.org.br; 6 Department of Clinical Genetics, Vejle Hospital, Institute of Regional Health Research, University of Southern Denmark, 7100 Vejle, Denmark

**Keywords:** dog, prostate cancer, proliferative inflammatory atrophy, microarray, copy number alteration, comparative oncology

## Abstract

Canine carcinomas have been considered natural models for human diseases; however, the genomic profile of canine prostate cancers (PCs) has not been explored. In this study, 14 PC androgen-receptor-negative cases, 4 proliferative inflammatory atrophies (PIA), and 5 normal prostate tissues were investigated by array-based comparative genomic hybridization (aCGH). Copy number alterations (CNAs) were assessed using the Canine Genome CGH Microarray 4 × 44K (Agilent Technologies). Genes covered by recurrent CNAs were submitted to enrichment and cross-validation analysis. In addition, the expression levels of *TP53*, *MDM2* and *ZBTB4* were evaluated in an independent set of cases by qPCR. PC cases presented genomic complexity, while PIA samples had a small number of CNAs. Recurrent losses covering well-known tumor suppressor genes, such as *ATM*, *BRCA1*, *CDH1*, *MEN1* and *TP53*, were found in PC. The in silico functional analysis showed several cancer-related genes associated with canonical pathways and interaction networks previously described in human PC. The *MDM2*, *TP53*, and *ZBTB4* copy number alterations were translated into altered expression levels. A cross-validation analysis using The Cancer Genome Atlas (TCGA) database for human PC uncovered similarities between canine and human PCs. Androgen-receptor-negative canine PC is a complex disease characterized by high genomic instability, showing a set of genes with similar alterations to human cancer.

## 1. Introduction

Prostate cancer (PC) is the second most common cancer in men worldwide and its incidence is especially high in developed countries [1]. Among mammals, the dog is the only animal that naturally develops prostate cancer with aging [2,3]. Moreover, by sharing the same environment, dogs have been considered a natural model for human cancer [4]. The canine disease usually displays a more aggressive behavior and is associated with a high metastatic rate and poor prognosis [3]. Furthermore, the continuous basal cell layer observed in the human prostate is not found in canine normal prostates, and no specific anatomic zones are associated with canine PC and prostatic hyperplasia development [5,6,7,8,9]. At the immunophenotypical level, canine PC commonly present loss of androgen receptor (AR), NKX3.1, and PTEN expression [10,11]. The expression levels of these three proteins are associated with the aggressive behavior of canine PC, and resembles the clinical features of human hormone-refractory PC [7]. Human PC is, androgen dependent, and only more aggressive cancers show loss of NKX3.1, and PTEN expression [12,13]. Canine PC can develop from luminal, ductal, and urothelial cells [3]. Therefore, a morphological analysis and a panel of proteins (uroplakin III, CK8/18, CK7, PSA, and AR) should be used to differentiate luminal and urothelial origins in tumors with undifferentiated patterns [7,14].

Despite all the differences, there are also some similarities at pathological, clinical, and molecular levels between canine and human PC. The concomitant presence of prostatic intraepithelial neoplasia, proliferative inflammatory atrophy (PIA), and bone metastases as well as the deregulation of cancer-related transcripts/proteins, including MDM2, PTEN, TP53, CTNNB1, and CDH1, are common events in dog and human PCs [10,11,15,16,17].

The first reports using comparative genomic hybridization (CGH) analysis to evaluate copy number alterations (CNAs) in dogs were performed in a glial tumor cell line and lymphomas [18,19]. The authors reported chromosomal imbalance similarities with human gliomas and lymphomas [18,19]. Subsequently, other neoplasms (transmissible venereal tumor, osteosarcoma, histiocytic sarcoma, and oral melanoma) were assessed through array-based CGH (aCGH), showing similar genomic alterations with the corresponding human tumors [20,21,22].

In canine PC, the cell line named Probasco, derived from a mixed breed dog with spontaneous tumors, presented genomic complexity, including large deletions, and duplications mapped in various chromosomes [23]. In this context, here, we report the genomic profile of canine pre-neoplastic prostate lesions and prostate carcinomas to better understand the molecular alterations involved in canine carcinogenic process. Moreover, three genes (*TP53*, *ZBTB4*, and *MDM2*) involved in regions with losses or gains were evaluated at the gene expression level. We also compared the genomic alterations in canine and human PCs. To our knowledge, this is the first study that has evaluated genomic imbalances in a cohort of dogs with spontaneous PC.

## 2. Results

### 2.1. Genomic Analysis

**Figure 1:**
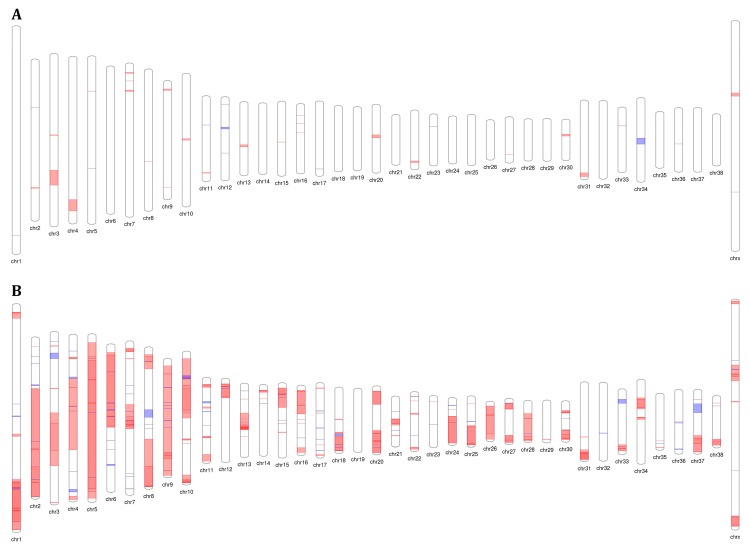
Representative copy number alterations (ideogram) showing gains (blue), and losses (red) in 4 proliferative inflammatory atrophies (PIAs) (**A**) and 14 canine prostate carcinomas (**B**).

The genomic profiling of 4 PIAs revealed 45 CNAs (40 gains, and 5 losses), with a mean of 11.25 alterations per case (Figure 1A). Twenty-two of 45 CNAs covered 37 known genes. Only LOC40355, mapped at the X chromosome, was altered in more than one case. Three CNAs (encompassing the *AZIN2*, *ADIPOQ*, *BCL6*, *CRYGS*, *MIR28*, *MIR8903*, and *SST* genes) were exclusively detected in PIA cases. Furthermore, 17 genes (*ACTG1*, *AK1*, *AKT2*, *BRD2*, *CD4*, *CRYAA*, *FAM83H*, *GNB3*, *LOC403555*, *MIR221*, *MIR338*, *SCN2B*, *SGSH*, *TIMP1*, *TNNT2*, *TPI1*, and *USP11*) were covered by CNAs in both PIA, and more than 20% of PC cases (≥3 cases). The detailed data are presented in Table S1.

A total of 370 CNAs (98 gains, and 272 deletions) were found in 14 PC samples. Particularly, three cases (8C, 8D1, and 40DM) presented more than 60 CNAs. Almost all chromosomes had genomic alterations, except chromosome 19 (Figure 1B). Losses of large segments were distributed along several chromosomes, particularly the chromosomes 2, 5, 6, 9, 10, 24, 26, and 28. Gains were also observed in numerous chromosomes but encompassing only small regions (details in Table S2). Moreover, 14 high copy gains (average log2 ratio > 2.0), and 12 homozygous deleted regions (average log2 ratio < −2.0) were observed in six and four cases, respectively. However, the majority of these CNAs covered no genes (Table S3).

**Figure 2:**
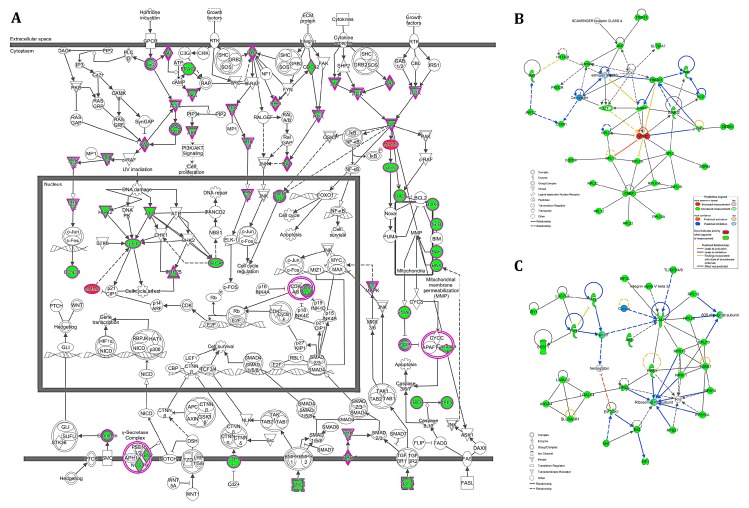
(**A**) Graphic representation of the canonical pathway called the molecular mechanism of cancer. Two cancer-related molecular interaction networks are indicated: (**B**) network 2, and (**C**) network 6, both associated with cell death and survival, organismal injury and abnormalities. Genes involved in gains, and losses are depicted in red and green colors, respectively. Images generated with the Ingenuity Pathway Analysis (IPA) software.

Recurrent alterations found in more than 20% of PC samples are depicted in Table S4. Likewise, 989 genes were affected by common CNAs (Table S5). Excluding the 221 microRNA genes, which involved one unique probe, 768 genes were evaluated using the Ingenuity Pathway Analysis (IPA) software. The in silico functional analysis revealed 655 of 768 (~85%) cancer-related genes (Table S6). Canonical pathway analysis identified several cancer-related pathways (Table S7), including molecular mechanisms involved with the disease (Figure 2A), and many specific signaling pathways in different types of cancer (e.g., colorectal, pancreatic, bladder, melanoma, breast, and prostate). Additionally, 5 of 25 networks were cancer-associated. Networks 2 (Figure 2B), and 6 (Figure 2C) are both associated with cell death and survival, organismal injury and abnormalities (Table S8).

**Figure 3:**
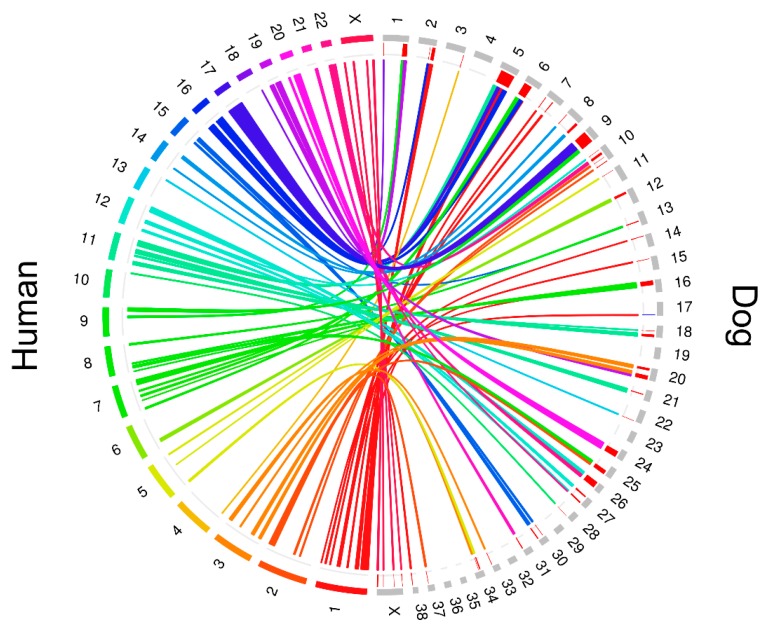
Circos plot showing recurrent copy number alteration (CNA) regions in canine prostate cancers (PCs), and the corresponding human chromosomal regions. The outer circle depicts canine (grey bars), and human chromosomes (colored bars). Regions of gains (blue), and losses (red) are shown between the outer and inner circles, below grey bars. The colored links indicate the corresponding regions involved in CNAs in dogs and humans.

The interspecies cross-validation analysis performed using human PC from The Cancer Genome Atlas (TCGA) database revealed 79 genes altered by genomic losses in both species (>20% of samples) (Table 1). The synteny map, constructed with CNAs detected in more than 20% of canine PC samples, shows the genomic similarities with human PC (Figure 3).

### 2.2. Transcript Expression Levels

**Figure 4:**
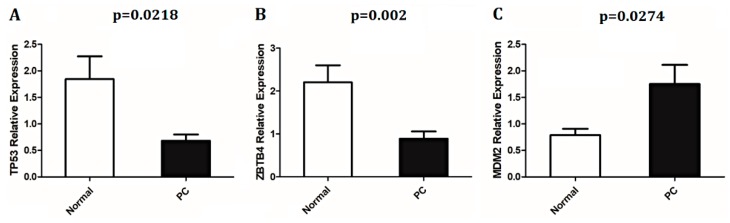
Relative expression (log scale) of *TP53* (**A**), *ZBTB4* (**B**), and *MDM2* (**C**) genes in normal and PC tissues. Data are presented as mean ± SD (standard deviation).

The qPCR analysis showed a significant down-expression of *TP53* (*p* = 0.0218; Figure 4A), and *ZBTB4* (*p* = 0.002; Figure 4B) in PC samples compared with normal prostate tissues. Significant increased *MDM2* transcript expression levels (*p* = 0.0274; Figure 4C) were observed in PC cases compared with normal tissues. The log2 ratio of *TP53*, and *ZBTB4* was −0.406666; −0.423839; −0.50188 (three cases presented both genes altered), and *MDM2* (three cases): 1.874149; 0.471251; 0.333158. 

## 3. Discussion

A comprehensive evaluation of canine cancer has the potential to uncover molecular mechanisms common to the human disease, including tumor progression, metastasis, and diagnostic, and prognostic markers. The presence of a high level of similarities opens avenues to identify combined or alternative therapies to better treat the disease in both species [24].

In a limited number of PIA cases studied, we detected a small number of CNAs, which revealed a low level of genomic instability. Among the seven genes exclusively identified in PIA, two (*ADIPOQ*, and *BCL6*) were previously described in human PC [25,26]. More interestingly, at least 11 (*AK1* silvia.regina.rogatto@rsyd.dk (S.R.R.) silvia.regina.rogatto@rsyd.dk (S.R.R.) silvia.regina.rogatto@rsyd.dk (S.R.R.) *AKT2*, *BRD2*, *CD4*, *FAM83H*, *GNB3*, *MIR221*, *MIR338*, *SCN2B*, *TIMP1*, and *TPI1*) of 17 genes affected by CNAs in PIA, and also PC samples were associated with different aspects of human prostate carcinogenesis (searched in the PubMed database using as headlines the gene name and prostate cancer, consulted in February 2019). A previous report using gene expression analysis showed that *TIMP* downregulation was implicated in the progression of prostatic intraepithelial neoplasia to PC [27]. In dogs, PIA is poorly explored and mostly limited to the immunohistochemistry (IHC) of a few proteins [11,17]. To our knowledge, this is the first study showing CNAs in primary canine PC, and PIA. Our findings point to a set of common genomic alterations in PIA, and PC, suggesting their involvement at the early stages of the disease. In human PIA, and prostatic intraepithelial neoplasia (PIN, a well-established preneoplastic lesion), similar frequencies of 8p22 loss, and 8q24 gain were described [28]. Apparently, several imbalances of chromosome 8 occur during human prostatic carcinogenesis [29]. Our results point out the presence of common chromosomal imbalances in canine PIA, and PC. Considering that PIA occurs at a higher frequency than in PIN [15], dogs could represent an interesting spontaneous model to study prostate cancer progression.

In contrast to PIA, PC presented several genetic alterations, predominately large deletions. The enrichment analysis revealed a large number of genes showing copy number alterations related to carcinogenesis. To our knowledge, only one immortalized canine prostate cancer cell line (Probasco) was characterized by CNAs [23]. Although this cell line presented various CNAs, similar to our cases, the authors found mainly gains, particularly of chromosomes 4, 6, 8, 10, 11, 14, 15, 17, 18, 20, 23, 24, 26, 35, 37, and 38 [23]. However, no comparison was performed between the primary tumor and the cell line. Although cell lines are excellent models to study several molecular aspects of cancer, it has been intensively discussed that culture conditions, cell growth time in vitro and cell immortalization can modify the DNA, and specific cells could be selected [30,31].

In human PC, chromosomal imbalances are prevalent and significantly more frequent than somatic mutations [32,33]. Deletions affecting tumor suppressor genes, including *BRCA1*, *BRCA2*, *CDH1*, and *TP53*, are common in human PC [34,35,36]. Similarly, our patients presented losses in several well-known tumor suppressor genes such as *ATM*, *BAX*, *BRCA1*, *CDH1*, *MEN1*, and *TP53*. Four homozygous deletions harboring genes (*LOC403555*, and *TNNT2*) were identified in three different PC cases. Among them, *LOC403555* has an uncertain function, and *TNNT2* is related to contraction of cardiac muscle cells [37].

Gains and amplifications have been associated with the activation of proto-oncogenes, which promote gain of function and contribute to cancer development or progression. We detected 14 high copy number gains and only 3 covered known genes (*QK1*, *BRD2*, and *EMR4P*). Among them, *BRD2* has been described as overexpressed in human castration-resistant PC [38]. Nevertheless, 6 of our 14 canine PCs also had hemizygous deletion of *BRD2*. In human PC cases from TCGA, CNAs mapped at *BRD2* are rare (8.3%; 41/492), being 18 gains, and 23 losses.

Interestingly, the synteny map showed similarities between recurrent losses detected in canine PCs with chromosomal losses described in human PCs, including 2q, 3p, 8p, 12p, 16q, 17p, and 21q.36. An interspecies cross-validation analysis using TCGA database, which currently contains 492 human PC cases with CNA information, revealed additional similarities between human and canine PC. At least 79 genes were altered by gains/losses in more than 20% of our cases and those from the TCGA. The inactivation of the clusterin gene (*CLU*), deleted in ~55% of TCGA cases, was associated with neoplastic transformation and tumor progression, while downregulation of *MX1* was involved in proliferation, progression and metastasis of PC cells [39,40,41]. Loss of the *ZBTB4* gene, a transcriptional repressor gene that binds to the GC-rich promoter regions of their targets, increases genomic instability (aneuploidy) and promotes tumorigenesis in human neoplasia, including PC [42,43]. In addition, *ZBTB4* overexpression is related to an increased survival time for PC-affected patients [44].

To better understand the relationship among the genes covered by CNAs in canine PC, we performed the canonical pathway and network analyses using the IPA software. The cancer-related canonical pathway called the molecular mechanisms of cancer revealed the involvement of genes reported as altered in several human cancers, including *TP53*, *MDM2*, *ATM*, and *BRCA1*. Additionally, network analysis revealed complex interactions among the genes. In particular, two networks directly related to cancer presented *MDM2*, *ICAM1*, and *FTH1* as central nodes. The deregulation of both *ICAM1*, and *FTH1* was previously associated with human prostate cancer [45,46]. The well-known *MDM2* oncogene is an important negative regulator of *TP53*, commonly deleted in human PC. Loss of *TP53* is involved with the transformation of prostate epithelium, and *MDM2* overexpression was associated with PC growth and metastasis [47,48]. Of interest, *ZBTB4* interacts both with *MDM2*, and *TP53* through *HIPK2*, a tumor suppressor involved in human cancer, and deleted in our cases [49,50].

Genomic alterations in *TP53*, *MDM2*, and *ZBTB4* were investigated at the transcriptional level in an independent cohort of PC patients. We found a significant down-expression of *TP53*, and *ZBTB4* in PC cases compared with normal tissues. In addition, *MDM2* was significantly overexpressed in cancer samples. Previously, we reported *TP53* down-expression and increased *MDM2* proteins, and transcript levels in a different cohort of animals with PC, thus confirming the results presented here [11]. Moreover, our data also suggested that these gene expression alterations might be, at least in part, explained by the presence of CNAs.

In addition to the similarities, we also observed differences between canine, and human PCs. Highly prevalent alterations in human PC, such as gains of the *MYC* oncogene and deletions of the tumor suppressors *NKX3-1*, *PTEN*, *RB1*, and *CDKN1B*, were absent or present in only one case [33,34]. While lacking studies of *RB1*, and *CDKN1B* in canine PC, expression of *MYC*, *NKX3-1*, and *PTEN* were assessed in our previous studies [11,35]. Using qPCR, IHC or Western blot, we reported *MYC* overexpression, and *NKX3-1*, and *PTEN* down-expression [11,35]. A plausible explanation is the sample bias or mechanisms other than CNAs involved in the *MYC* activation (translocation or mutation), and *PTEN*/*NKX3-1* inactivation (point mutations or hypermethylation).

An interesting difference between canine and human PC relates to the AR. AR has been described as involved in the development, progression and survival of human PC cells [50,51]. In human PC, AR is an oncogenic driver and its amplification/overexpression has been suggested as a mechanism that leads to the appearance of castration-resistant PC, which hypersensitizes cancerous cells to low levels of androgens [52,53]. In our PC sample set, genomic loss involving the AR gene was observed in three cases and no gains or amplifications were detected. Canine PCs have been characterized by a lack of or low AR protein levels, and the animals are not responsive to castration as a therapeutic option [7,11,54,55]. Previous reports have suggested that the initial transformation of neoplastic canine prostate cells starts from an AR-negative clone, even in intact animals [7,11].

All canine PC AR-negative cases herein evaluated by CNAs presented a Gleason score of 6 and no metastasis. In addition, no information on AR protein expression in TCGA dataset was available, thus limiting a more detailed comparison. Although these factors are limitations of our study, we reported similar genomic alterations in both human and canine PC, addressing a gap in the literature. Further studies are needed to unravel genes and pathways involved in more aggressive canine PC, including in metastatic cases.

## 4. Materials and Methods

### 4.1. Ethics Statement

This study was performed in accordance to the National and International Recommendations for the Care and Use of Animals. All procedures were performed under the approval of the Animal Ethics Committee from Faculty of Veterinary Medicine and Animal Science, UNESP, Botucatu, SP, Brazil (Protocol #10/2007).

### 4.2. Patients

The total number of cases included in this study was 33 PCs, 4 PIAs, and 15 normal prostate tissues. Array-CGH assays were performed in 14 PC, 4 PIA, and 5 normal prostatic tissues. The necropsies were performed within an interval of 1 h from the animal’s death, with the consent of the owners. The mixed breed dogs were intact and had a mean age of 9 years. Although they had no clinical features of PC or PIA, lesions were found in the histopathological analysis. The prostatic gland was collected, fixed in 10% formalin, routinely processed, embedded in paraffin and sectioned. The areas of interest were identified and the cells were obtained from macrodissected samples using a 16-gauge needle, as previously described [11]. Tumor areas with more than 85% of epithelial neoplastic cells were selected for DNA extraction (Figure S1). The PIA cases evaluated in our study were diagnosed during necropsies and no evidence of PC was found in these animals. The histopathological characterizations of PIA, and PC samples were performed according to Palmieri et al. [15], and Lai et al. [56], respectively. All PC cases presented an infiltrative pattern with no measurable mass and the same tumor grade (Gleason score of 6). In this set of cases, no metastasis was found during the necropsies. Furthermore, all PC samples showed negativity for uroplakin III immunoexpression [7].

An independent formalin-fixed paraffin-embedded (FFPE) sample set composed by 19 PC (biopsy samples), and 10 normal prostate tissues (necropsy samples) were used to evaluate the gene expression levels (*TP53*, *MDM2*, and *ZBTB4*) by qPCR. The mean age was 10.2 years (8–15 years), and the mean survival time was 237.6 days (12–523 days). All 19 PC patients were evaluated by X-ray, and computerized tomography (CT) for screening of metastasis at the diagnosis. Thirteen of them (13/19) had metastatic disease at diagnosis in multiple sites including bone (7/13), lung (5/13), intestine (2/13), liver (1/13), and iliac lymph node (1/13). All PC cases were negative for androgen receptor immunostaining. In this cohort of 19 PC cases, 13 presented a Gleason score of 10, 6 a Gleason score of 6 and 3 a Gleason score of 8. The metastatic cases presented Gleason scores of 10 (10 of 13 cases) and 8 (3 of 13) (Figure S1).

### 4.3. Chromosomal Imbalances Analysis (aCGH)

Genomic DNA was extracted from FFPE samples using the Qiagen DNeasy Blood & Tissue Kit (Quiagen, Hilden, Germany) according to the manufacturer’s recommendations. A pool of genomic DNA obtained from five FFPE canine normal prostate tissues was used as reference.

Copy number alterations were assessed using the Canine Genome CGH Microarray 4 × 44K G2519F (ID-021193, Agilent Technologies, Santa Clara, CA, USA), according to the manufacturer’s instructions. Data were extracted with Feature Extraction 10.1.1.1 software (Agilent), and the analyses were performed using Agilent Genomic Workbench Standard Edition 5.0.14 software (Statistical algorithm ADM-2, threshold of 4.0, fuzzy zero correction). Nonredundant CNAs called by at least three consecutives probes and a log2 ratio > 0.3 for gains and <−0.3 for losses were considered as significant. The ideograms showing the CNAs identified in each case were constructed using the PhenoGram online software (http://visualization.ritchielab.psu.edu/phenograms/document).

Synteny blocks between canine and human genomes were obtained through the Synteny Portal [57]. The SynBuilder tool was used to obtain synteny blocks between the canine canFam2 and human hg19 reference genomes, with a resolution of 150 Kbp. Regions overlapping CNAs in more than 20% of PC cases were identified with the CoNVaQ web tool [58]. The Circos plot showing the altered regions and the corresponding regions in humans was generated using the OmicCircos at R/Bioconductor package [59].

Enrichment analysis, based on human data, was performed with Ingenuity Pathway Analysis software (IPA v.2.2.1, Qiagen, http://www.qiagen.com/ingenuity/), considering the genes affected by CNAs in more than 20% of PC cases. In this analysis, the following parameters were used: core analysis, Ingenuity Knowledge Base (genes only) as a reference set, direct and indirect relationships, endogenous chemicals, 35 molecules per network and a maximum of 25 networks. Statistical significance was considered with *p* < 0.05 calculated using Fisher’s exact test. The activation (orange lines in the network) or inhibition (blue lines in the network) was used with z-score × 2.0 or ≤ −2.0, respectively. The CNA information of 492 human PCs from TCGA database, stored in the cBioPortal for Cancer Genomics (http://www.cbioportal.org/, accessed in April 2018), was used to explore the similarities between canine and human PCs.

### 4.4. Gene Expression Analysis

A hematoxylin-eosin tissue section (PC, and normal tissue) slide to guide the tissue dissection was demarcated and isolated using a 40-gauge needle in the FFEP block. Total mRNA was extracted using the RecoverAll™ Total Nucleic Acid Kit (Ambion, Life Technologies, MA, USA), according to the manufacturer’s instructions. cDNA synthesis was carried out as previously described [11]. The quantity and quality of RNA were evaluated using a NanoDrop ND-1000 Spectrophotometer v.3.0.1 (Labtrade, Wilmington, NC, USA), and 2100 Bioanalyser RNA 6000 Nano kit (Agilent Technologies, Palo Alto, CA, USA), respectively.

Three genes (*TP53*, *MDM2*, and *ZBTB4*) were selected and evaluated by qPCR based on their importance in carcinogenesis, network analysis and TCGA cross-validation analysis. *TP53*, and *MDM2* are well-known cancer genes involved in the emergence of several human malignancies, including PC. *TP53* has been implicated in several cancer-related canonical pathways, and *MDM2* is a central node in the molecular network 2. In addition, *ZBTB4* indirectly interacts with MDM2, and has also been related to prostate carcinogenesis. Moreover, *TP53*, and *ZBTB4* deletions were cross-validated using TCGA database analysis.

The primer sequences of *TP53*, *MDM2*, and reference genes (*ACRB*, *HPRT*, and *PRS5*) were previously published [11,60]. The primer sequences of *ZBTB4* was forward: 5′TTGGGATTTTGCCATTTCG 3′, and reverse: 5′GGGCAAGGAGGGCACAA 3′. qPCR for *TP53*, *MDM2*, *ZBTB4*, and the endogenous genes was conducted in a total volume of 10 μL containing Power SYBR Green PCR Master Mix (Applied Biosystems; Foster City, CA, USA), 1 μL of cDNA (1:10), and 0.3 μM of each primer. The reactions were performed in triplicate in 384-well plates using QuantStudio 12K Flex Thermal Cycler equipment (Applied Biosystems, Foster City, CA, USA). The PCR product specificity was determined by dissociation curve for all experiments. Relative gene expression was quantified using the 2^−ΔΔCT^ method [61].

### 4.5. Statistical Analysis

Statistical analysis was performed using GraphPad Prism 5 v.5.0 (GraphPad Software Inc., La Jolla, CA, USA). The Kruskal–Wallis or Mann–Whitney U test was applied to compare *TP53*, *MDM2*, and *ZBTB4* transcript levels between normal and PC samples. Statistical significance was set at *p* < 0.05.

## 5. Conclusions

To our knowledge, this is the first study to describe the genomic profile in a cohort of canine PIA, and PC samples. Our results revealed that PIA cases presented a small number of CNAs compared with PC. PC samples presented a large number of chromosomal imbalances, mainly deletions, which revealed a set of cancer-related genes involved in canonical pathways or networks previously described in human PC. Deletions of *TP53*, and *ZBTB4*, and gains of *MDM2* were translated in down- and overexpression, respectively, at the transcriptional level. Furthermore, TCGA cross-validation analysis pointed out similarities between human and canine prostate carcinogenesis. Canine PC is an interesting model to use in comparative prostate cancer research; however, it is important to note the differences, including those reported for the AR gene.

## Figures and Tables

**Table 1:** List of Genes Altered by CNAs in More than 20% of Our PC Cases (*N* = 14), and in The Cancer Genome Atlas (TCGA) Dataset (*N* = 492).

COPY NUMBER LOSSES					
Gene	Canine PC (*N* = 14)	Human PC TCGA (*N* = 492)	Gene	Canine PC (*N* = 14)	Human PC TCGA (*N* = 492)
*ACTB*	4	7	*MTPN*	3	12
*ADRA1A*	3	275	*MX1*	5	134
*AZGP1*	4	7	*MX2*	5	130
*CASP2*	3	11	*MYH1*	4	110
*CCL17*	3	123	*MYH2*	3	110
*CCL24*	4	5	*MYH3*	3	109
*CCL26*	4	3	*MYH4*	4	110
*CDH1*	3	143	*MYH8*	4	110
*CFDP1*	3	187	*NIP7*	3	146
*CHST4*	3	169	*NOS3*	3	12
*CLCN1*	3	11	*OCLN*	2	102
*CLDN3*	4	3	*PDGFA*	4	13
*CLU*	3	268	*PLA2G15*	3	136
*CNGB1*	3	118	*POR*	4	4
*CX3CL1*	3	123	*RAC1*	4	7
*CYBA*	3	192	*RCVRN*	3	110
*EIF2AK1*	4	7	*RPL26*	3	119
*EIF4A1*	3	159	*SERPINE1*	4	10
*EPO*	4	6	*SLC12A4*	3	132
*FAM83H*	8	12	*SLC2A4*	3	155
*FASTK*	3	12	*SLC4A1*	3	96
*GALNS*	3	192	*SLC7A5*	3	202
*GP1BA*	3	103	*SSBP1*	3	9
*GUCY2D*	3	153	*SSPO*	3	10
*HIPK2*	3	10	*TAS2R3*	3	9
*HSF4*	4	130	*TAS2R38*	3	12
*HSPB1*	3	3	*TAS2R39*	3	11
*KCNH2*	3	12	*TAS2R40*	3	11
*LUC7L2*	3	9	*TAS2R41*	3	10
*MC1R*	3	188	*TAS2R5*	3	9
*MIR106B*	4	7	*TEKT1*	3	131
*MIR140*	3	152	*TNFSF12*	3	159
*MIR25*	4	7	*TNFSF13*	3	159
*MIR324*	3	149	*TP53*	3	163
*MIR328*	3	130	*TRPV1*	3	93
*MIR490*	3	10	*WWP2*	3	153
*MIR589*	4	7	*ZBTB4*	3	158
*MIR590*	4	4	*ZNF252*	4	13
*MIR671*	3	12	*ZP3*	3	5
*MIR93*	4	7			

## Supplementary Materials

Supplementary Materials can be found at https://www.mdpi.com/1422-0067/20/7/1555/s1. Figure S1: Histopathology pattern of the canine prostate cancer (PC) samples according to the Gleason score. (A) PC Gleason score 6, showing a well-differentiated tubular formation (arrows). (B) PC Gleason score 8, presenting a high proliferation of tumor cells (arrows) and only few remaining tubular formations. (C) PC Gleason score 9 presenting a cribriform morphology. (D) PC Gleason score 10 showing solid pattern, Table S1: List of genomic alterations identified in four PIA cases, Table S2: List of genomic alterations identified in 14 PC cases, Table S3: List of high copy gains and homozygous deleted regions found in the 14 PC samples, Table S4: Recurrent CNAs in canine PCs (present in more than 20% of cases), Table S5: List of genes covered by CNAs in more than 20% of PC cases, Table S6: Cancer-related functions of genes evaluated by IPA software, Table S7: Cancer-related canonical pathways of genes evaluated by IPA software, Table S8: Gene interaction networks generated by IPA software.

## Abbreviations

PC	Prostate cancer
PIA	Proliferative inflammatory atrophy
CNAs	Copy number alterations
TCGA	The Cancer Genome Atlas
CGH	Comparative genomic hybridization
aCGH	Array-based comparative genomic hybridization
IHC	Immunohistochemistry
IPA	Ingenuity Pathway Analysis
PIN	Prostatic intraepithelial neoplasia
