# Supplementary material for: Comprehensive Genomic Profiling of Androgen-Receptor-Negative Canine Prostate Cancer
Source: Int J Mol Sci. 2019 Mar 28;20(7):1555. doi: 10.3390/ijms20071555 (PMC6480132; doi:10.3390/ijms20071555)

**Supplementary figure 1.** Histopathology pattern of the canine prostate cancer (PC) samples according to the Gleason score. (**A**) PC Gleason score 6, showing a well-differentiated tubular formation (arrows). (**B**) PC Gleason score 8, presenting a high proliferation of tumor cells (arrows) and only few remaining tubular formations. (**C**) PC Gleason score 9 presenting a cribriform morphology. (**D**) PC Gleason score 10 showing solid pattern.


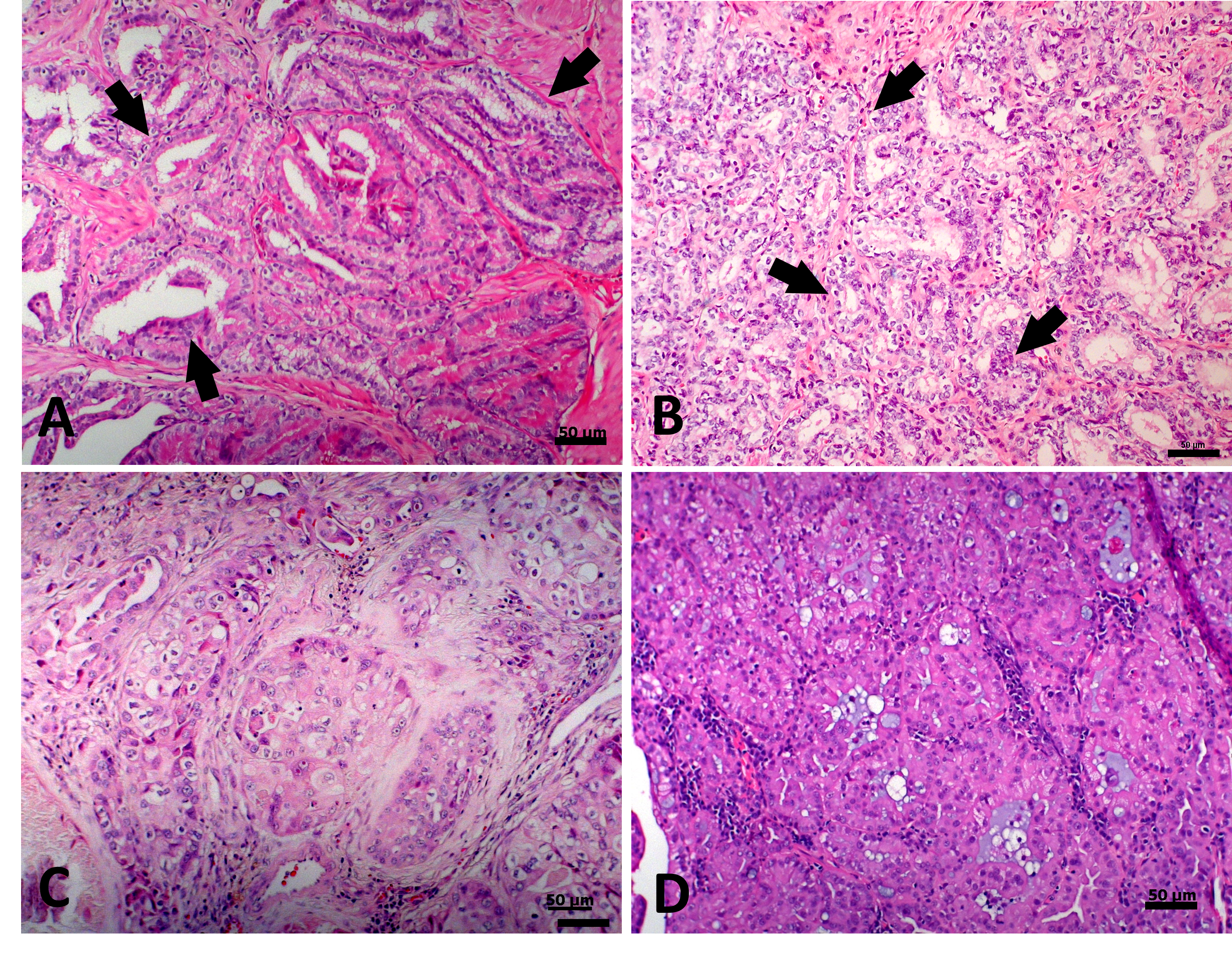

Supplement: Supplementary file 1 [file ijms-20-01555-s001.zip › Supplementary figure 1.docx]
